# Trainable Quaternion Extended Kalman Filter with Multi-Head Attention for Dead Reckoning in Autonomous Ground Vehicles

**DOI:** 10.3390/s22207701

**Published:** 2022-10-11

**Authors:** Gary Milam, Baijun Xie, Runnan Liu, Xiaoheng Zhu, Juyoun Park, Gonwoo Kim, Chung Hyuk Park

**Affiliations:** 1 Department of Biomedical Engineering, George Washington University, Washington, DC 20052, USA; 2 Korea Institute of Science and Technology, Seoul 02792, Korea; 3 Department of Control and Robot Engineering, ChunBuk National University, Chungbuk 28644, Korea

**Keywords:** localization, inertial navigation system, extended Kalman filter, mobile robot, autonomous ground vehicle

## Abstract

Extended Kalman filter (EKF) is one of the most widely used Bayesian estimation methods in the optimal control area. Recent works on mobile robot control and transportation systems have applied various EKF methods, especially for localization. However, it is difficult to obtain adequate and reliable process-noise and measurement-noise models due to the complex and dynamic surrounding environments and sensor uncertainty. Generally, the default noise values of the sensors are provided by the manufacturer, but the values may frequently change depending on the environment. Thus, this paper mainly focuses on designing a highly accurate trainable EKF-based localization framework using inertial measurement units (IMUs) for the autonomous ground vehicle (AGV) with dead reckoning, with the goal of fusing it with a laser imaging, detection, and ranging (LiDAR) sensor-based simultaneous localization and mapping (SLAM) estimation for enhancing the performance. Convolution neural networks (CNNs), backward propagation algorithms, and gradient descent methods are implemented in the system to optimize the parameters in our framework. Furthermore, we develop a unique cost function for training the models to improve EKF accuracy. The proposed work is general and applicable to diverse IMU-aided robot localization models.

## 1. Introduction

Over the past years, localization has become one of the challenging issues for autonomous ground vehicles (AGVs). In particular, the most difficult issue in navigation is estimating the accurate and stable position and orientation of the robot through data obtained from sensors and other navigation systems [1]. Recently, various technologies have been designed to solve robot localization problems, such as visual-odometry-aided camera localization [2] and Global Positioning System (GPS)-based localization using reinforcement learning [3].

However, the simultaneous localization and mapping (SLAM) methods may fail to function correctly in certain complicated situations due to the physical characteristics of sensors. The laser imaging, detection, and ranging (LiDAR) sensor, for example, is a sensor system that measures distance by transmitting light into spaces and receiving reflected signals from a target [4]. However, LiDAR can lose its signal in situations such as foggy and rainy conditions. In addition to that, the strength of the signal can be affected by the reflectivity of the objects. This could lead a mobile robot or an AGV into a target-blind zone, endangering safety and maneuverability. Therefore, a reliable contingency plan needs to be considered that can compensate for the performance degradation caused by these limitations of the sensors.

The inertial measurement unit (IMU), a combination of a gyroscope, accelerometers, and sometimes magnetometers, could provide an efficient approach to solve this problem. Specifically, the IMU is one of the solutions that can independently measure the state of the body without any external feedback. The accelerometer and gyroscope components measure linear acceleration and angular velocity, which represent the movement of the body to which the sensor is attached. Additionally, the magnetometer component measures orientation based on the Earth’s magnetic field, which is available almost everywhere [5].

Nevertheless, there is one drawback that could degrade the performance of an IMU-based localization, which is the accumulated drift. To reliably utilize the IMU, it is imperative to eliminate the accumulated drift [1]. In the field of probabilistic robotics, various methods for correcting errors, such as Bayes filters, Gaussian filters (e.g., information filters), and nonparametric filters (e.g., particle filters) [6], have been proposed. Among them, the extended Kalman filter (EKF) [7] is one of the most widely used methods to reduce the accumulated drift. The EKF is a nonlinear Kalman filter (KF) that linearizes a current mean and covariance estimate. Since EKF can solve nonlinear problems, it has been applied to IMU-aided localization systems [8,9,10]. The process-noise covariance matrix, *Q*, and the measurement-noise covariance matrix, *R*, are constructed with a priori constant values determined by the characteristics of sensors and environments in traditional KF systems, which assume that they remain constant throughout the whole navigation operation. EKF can achieve optimal results if the process noise is well defined. However, depending on external factors, such as complex environments or sensor limitations (e.g., occlusions), sensor noise values can change, and it is difficult to recognize the exact error and in situ information of when and how the change occurs [11].

The following is a list of the major contributions of this study:

1. In this work, we propose a novel approach to improve the accuracy of EKF-based IMU localization with a convolutional neural network (CNN) architecture. Specifically, we design a stable training method that can find the optimal parameters of the system and the observation-noise covariance in real time by reducing the error in each iteration. Furthermore, the system is designed and tested for online training, unlike many other approaches, such as [12], where the algorithm is trained offline using batch and multiple epochs. The intention behind this is that the algorithm is to be trained continuously while SLAM is functioning online, in which case a sequence of IMU data points is observed and acquired.
2. Our proposed CNN module consists of multi-head attention (MHA) layers to model the cross-modal fusion of different sources of modalities (e.g., multiple IMUs, lidars, etc.). The MHA was initially proposed to address the problems of natural language processing (NLP) [13], and it was later discovered to be effective in modeling cross-modal interactions between different modalities [14]. These previous works inspired us to model cross-modal interactions that combine different sensor information sources via the attention mechanism.
3. We conducted extensive experiments using an actual robotic platform to assess the effectiveness of our proposed method in the real world (a factory environment in our case). We designed real-world scenarios for the online training, where the SLAM might fail in some cases and only the IMU(s) can provide sensory information for the EKF-based localization module. The algorithm is also trained continuously while the robot is online and navigating.

## 2. Related Work

Studies have been conducted recently on Kalman filter (KF)-based localization technology with adaptive noise-covariance estimation. One previous study proposed by Akhlaghi et al. [15] introduced innovation-based and residual-based methods to adaptively adjust the covariance matrices *Q* and *R* at each step of the EKF process to improve the state estimation accuracy. In addition, Hu et al. [16] proposed an adaptive unscented Kalman filter (UKF), another variant of KF for the nonlinear system, with process-noise covariance estimation to improve the UKF performance. However, these approaches might not be able to fully characterize the nonlinear stochastic noises that arise in real-world situations.

It is known that the artificial neural network has the capability to approximate nonlinear functions [17]. Haarnoja et al. [18] demonstrated that a backpropagation-based Kalman filter, consisting of a KF and a CNN, was capable of predicting the measurement-noise covariance matrix, where the CNN was trained via minimizing position errors. Brossard et al. [12] proposed an approach for dead-reckoning for wheeled vehicles with the IMU only. Deep neural networks were used to update the parameters of an invariant EKF dynamically. A recent study also explored the use of long short-term memory (LSTM), a type of recurrent neural network (RNN), to model the nonlinear noises for KF [19] to address target tracking problems. Another approach that uses reinforcement learning to adaptively estimate the process-noise covariance matrix was proposed by Gao et al. [20], in which their algorithm used the deep deterministic policy gradient (DDPG) to extract the optimal process-noise covariance matrix estimation from the continuous action space, using an integrated navigation system as the environment and the reverse of the current positioning error as the reward. Wu et al. [21] also proposed a deep learning framework combining a denoising autoencoder and a multitask temporal CNN. Multitask learning was used to optimize the loss for both the process-noise covariance and measurement-noise covariance matrices from KF simultaneously.

## 3. Quaternion-Based Extended Kalman Filter

### 3.1. IMU Inclination Calculation

The rotation matrix $R_{n}^{b}$, mapping the navigation frame *n* to the body frame *b*, can be represented by ϕ (rotation angle along the x-axis), θ (rotation angle along the y-axis), and ψ (rotation angle along the z-axis), as follows (trigonometric functions sin and cos are denoted as *s* and *c*, respectively):

(1) $$\begin{array}{c} R_{n}^{b}=\left[\begin{array}{ccc} c\theta c\psi & c\theta s\psi & -s\theta \\ c\psi s\theta s\phi -c\phi s\psi & c\phi c\psi +s\theta s\phi s\psi & c\theta s\phi \\ c\phi c\psi s\theta +s\phi s\psi & c\phi s\theta s\psi -c\psi s\psi & c\theta c\phi \end{array}\right]. \end{array}$$

When the IMU is stationary or moving at a constant speed, the acceleration in the navigation frame should be equal to the gravity constant *g*, so the inclination angles θ and ϕ can be calculated by Equation (2) as in [22]:

(2) $$\left[\begin{array}{c} a_{x}^{b}\\ a_{y}^{b}\\ a_{z}^{b} \end{array}\right]=R_{n}^{b}\left[\begin{array}{c} 0\\ 0\\ g \end{array}\right]\;\Rightarrow \;\left\{\begin{array}{c} \theta =\arcsin(-\frac{a_{x}^{b}}{g})\\ \phi =\arctan(\frac{a_{y}^{b}}{a_{z}^{b}}) \end{array}\right..$$

**Figure 1:**
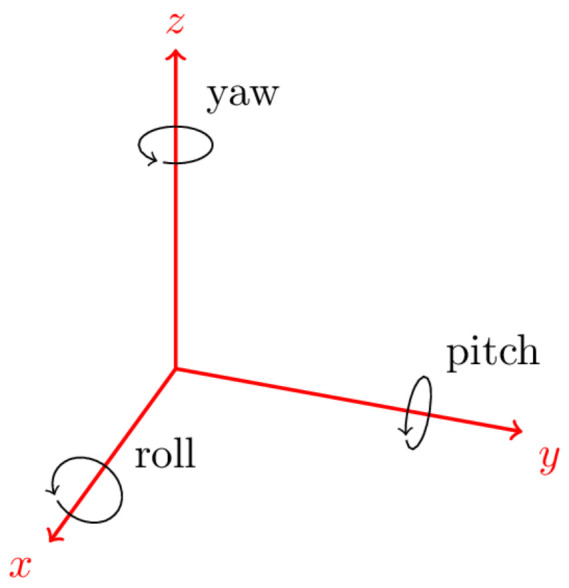
The IMU coordinate frame.

The IMU coordinate frame is assumed as shown in Figure 1, where the z-axis is upward, so gravity has a positive value.

### 3.2. IMU Integration Model

The continuous-time relationships among position $p^{n}$, velocity $v^{n}$, and acceleration $a^{n}$ are defined as follows:

(3) $$\frac{\partial p_{t}^{n}}{\partial t}=v_{t}^{n},\;\frac{\partial v_{t}^{n}}{\partial t}=a_{t}^{n},$$

where $a_{t}^{n}$ represents the acceleration in the navigation frame at time *t*, which can be calculated using $a_{t}^{b}$, which is the acceleration obtained from the IMU sensor as follows:

(4) $$a_{t}^{n}=R\left(q_{b}^{n}\right)a_{t}^{b}-g^{n},$$

where $R(q_{b}^{n})$ is the rotation matrix represented by quaternion $q_{b}^{n}$. The orientation $q_{b}^{n}$ and the angular velocity $\omega^{b}$ are related as

(5) $$\frac{\partial q_{b}^{n}}{\partial t}=q_{b}^{n}⊙\frac{1}{2}\omega^{b}.$$

From the continuous-time model, the dynamics of position, velocity, and orientation in discrete time are given by Equations (6)–(8), as explained in [23], as follows:

(6) $$p_{t}^{n}=p_{t-1}^{n}+v_{t-1}^{n}\cdot \delta t+\frac{1}{2}\left(a_{t-1}^{n}+e_{a,t}\right)\cdot \delta t^{2}$$

(7) $$v_{t}^{n}=v_{t-1}^{n}+\left(a_{t-1}^{n}+e_{a,t}\right)\cdot \delta t$$

(8) $$q_{b,t}^{n}=q_{b,t-1}^{n}⊙exp_{q}\left(\frac{1}{2}\left(\omega_{t-1}^{b}-e_{\omega ,t}\right)\cdot \delta t\right),$$

where $e_{a,t}$, $e_{\omega ,t}$ are the noise terms of the dynamics model which are assumed to follow the normal distribution, and the distribution axes are independent of each other, as follows:

(9) $$\begin{array}{cc} \; & e_{a,t}∼N\left(0,\sigma_{a}I_{3}\right) \end{array}$$

(10) $$\begin{array}{cc} \; & e_{\omega ,t}∼N\left(0,\sigma_{\omega }I_{3}\right). \end{array}$$

The state variable $x_{t}$ is a 10×1 vector consisting of the current position $p_{t}^{n}$, velocity $v_{t}^{n}$, and orientation $q_{b,t}^{n}$ which represents the mapping of the body frame onto the navigation frame, as follows:

(11) $$x_{t}={\left[\begin{array}{ccc} p_{t}^{n}, & v_{t}^{n}, & q_{b,t}^{n}, \end{array}\right]}_{10\times 1}^{T}$$

so the state transition function can be written as

(12) $${\hat{x}}_{t|t-1}=f\left({\hat{x}}_{t-1|t-1},u_{t},e_{t}\right),$$

where $u_{t}={\left[a_{t}^{b},\;\omega_{t}^{b}\right]}^{T}$ is the control input modeled by the accelerometer and gyroscope measurements, and the noise term $e_{t}={\left[e_{a,t},\;e_{\omega ,t}\right]}^{T}$.

We linearize Equation (12) at the current estimate and propagate the covariance forward to predict the system covariance

(13) $$P_{t|t-1}=F_{t}P_{t-1|t-1}F_{t}^{T}+G_{t}QG_{t}^{T},$$

where $F_{t}$, $G_{t}$ are Jacobian matrices of the transition function (12) with respect to $x_{t}$ and $u_{t}$, as shown below:

(14) $${\left.F_{t}=\frac{\partial f(x_{t},u_{t},e_{t})}{\partial x_{t}}\right|}_{\begin{array}{l} e_{t}=0\\ x_{t}={\hat{x}}_{t-1|t-1} \end{array}}$$

(15) $${\left.G_{t}=\frac{\partial f(x_{t},u_{t},e_{t})}{\partial u_{t}}\right|}_{\begin{array}{l} e_{t}=0\\ x_{t}={\hat{x}}_{t-1|t-1} \end{array}},$$

and the process covariance $Q=diag(\Sigma_{a},\;\Sigma_{a},\;\Sigma_{\omega })$, where $\Sigma_{a}$ and $\Sigma_{\omega }$ represent the covariances of acceleration and angular velocity, respectively.

### 3.3. EKF Correction with Measurements

Since the accelerometer measures the local gravity vector when an AGV is moving at a constant speed or is stationary, it can provide information about the inclination of the sensor [23]. Then, the robot control center can provide the velocity information $v^{b}$ along the x-axis and y-axis at current times. In addition, we consider the vertical velocity, which is roughly null in the robot frame, as a pseudo-velocity measurement $v_{pseudo}^{b}$, so the total measurement vector can be written as

(16) $$z_{t}={\left[\begin{array}{ccc} a_{t}^{b}, & v_{cmd,t}^{b}, & v_{pseudo,t}^{b}=0 \end{array}\right]}_{6\times 1}^{T}.$$

Thus, we can obtain the measurement function mapping the state space to the measurement space as follows:

(17) $$h\left({\hat{x}}_{t|t-1}\right)=\left[\begin{array}{c} R{\left({\hat{q}}_{b,t|t-1}^{n}\right)}^{T}g^{n}\\ R{\left({\hat{q}}_{b,t|t-1}^{n}\right)}^{T}{\hat{v}}_{t|t-1}^{n} \end{array}\right],$$

Then, we obtain the measurement matrix $H_{t}$ by linearizing the measurement function:

(18) $${\left.H_{t}=\frac{\partial h(x_{t})}{\partial x_{t}}\right|}_{x_{t}={\hat{x}}_{t|t-1}}.$$

Therefore, the measurement residual $y_{t}$ and the Kalman gain $K_{t}$ are calculated by Equations (19) and (20), as detailed in [24]:

(19) $$y_{t}=z_{t}-h\left({\hat{x}}_{t|t-1}\right)$$

(20) $$K_{t}=P_{t|t-1}H_{t}^{T}{\left(H_{t}P_{t|t-1}H_{t}^{T}+R\right)}^{-1},$$

where the measurement covariance matrix R can be defined as $diag(\Sigma_{a},\;\Sigma_{v},\;\Sigma_{v_{pseudo}})$. Finally, the predicted state and covariance are updated as follows:

(21) $$\begin{array}{cc} \; & {\hat{x}}_{t|t}={\hat{x}}_{t|t-1}+K_{t}y_{t}, \end{array}$$

(22) $$\begin{array}{cc} \; & P_{t|t}=\left(I-K_{t}H_{t}\right)P_{t|t-1}. \end{array}$$

## 4. Covariance Optimization

### 4.1. Adjustable Covariance

It is well known that the EKF is a model-based optimal filter, which requires exact knowledge of process and measurement models as well as process- and measurement-noise statistics. However, it is difficult to model the dynamic noise changes over time [25]. Thus, we redesign the covariance Σ of the covariance matrices Q and R as follows:

(23) $$\begin{array}{cc} \Sigma_{a} & =\sigma_{a}^{2}\cdot 10^{\mu \tanh(s_{a})} \end{array}$$

(24) $$\begin{array}{cc} \Sigma_{\omega } & =\sigma_{\omega }^{2}\cdot 10^{\mu \tanh(s_{\omega })} \end{array}$$

(25) $$\begin{array}{cc} \Sigma_{v_{cmd}} & =\sigma_{v_{cmd}}^{2}\cdot 10^{\mu \tanh(s_{v_{cmd}})} \end{array}$$

(26) $$\begin{array}{cc} \Sigma_{v_{pseudo}} & =\sigma_{v_{pseudo}}^{2}\cdot 10^{\mu \tanh(s_{v_{pseudo}})}, \end{array}$$

where $\sigma_{a}$, $\sigma_{\omega }$, $\sigma_{v_{cmd}}$, and $\sigma_{v_{pseudo}}$ correspond to our initial estimate of the noise parameters and μ>0. Thus, the covariance can be limited between a factor $10^{-\mu }$ and a factor $10^{\mu }$ with respect to its original value because of the function tanh(·), which makes the covariance optimized within a reasonable interval set heuristically. By adjusting the value of the parameter *s*, the covariance matrices could be changed indirectly.

Parameters $s_{a}$ and $s_{\omega }$ are adjusted during training by backpropagation based on the loss function described by Equation (30). Once the training stops, the parameters are considered fixed for the algorithm.

Although the noise components of the velocity *v* and pseudo-velocity $v_{pseudo}$ are unknown, the deviation can be assumed to be dynamic rather than stationary in the real world. In other words, the measurement covariance from velocity can be treated as loose strict null instead of strict null, which means that the uncertainty can be encoded in the covariance [12]. A CNN layer is applied to dynamically compute the parameters $s_{v}$ and $s_{v_{pseudo}}$, taking as input a window size of N IMU data points.

(27) $$\left[\begin{array}{c} s_{v}\\ s_{v_{pseudo}} \end{array}\right]=\left[\begin{array}{c} \left[\begin{array}{c} s_{v_{x}}\\ s_{v_{y}} \end{array}\right]\\ s_{v_{pseudo}} \end{array}\right]=CNN\left(\left[u_{t-N},\;\ldots u_{t}\right]\right).$$

**Figure 2:**
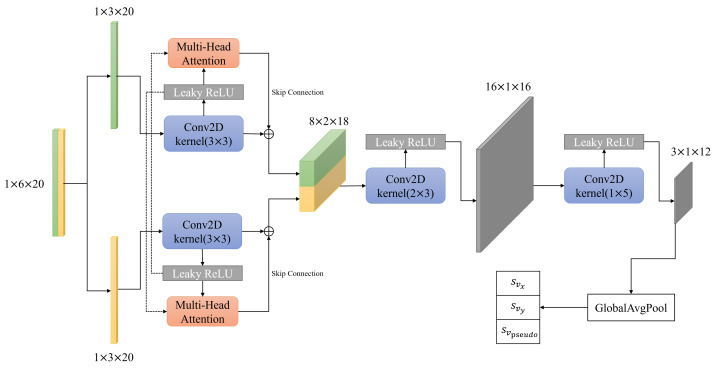
The proposed CNN architecture for predicting the parameters $s_{v_{x}}$, $s_{v_{y}}$, and $s_{v_{pseudo}}$, which are used to calculate the covariances as per Equations (25) and (26). The input consists of a window size of 20 IMU data points, each containing the acceleration and angular velocity data for all three axes.

Figure 2 shows the CNN architecture used to predict parameters $s_{v}$ and $s_{v_{pseudo}}$. A window size of 20 IMU data points was used for the input. Each IMU data point consists of acceleration and angular velocity data for the three axes (*x*,*y*,*z*). Using a single channel, where the overall input dimension becomes 1×6×20, the input is initially split into its individual acceleration and angular velocity matrices, which are processed separately by their respective 3×3 convolution layers followed by a leaky ReLU activation (ConvLR block). Then, a multi-head attention (MHA) layer is introduced to model the feature-fusion data. The output of the individual paths is then concatenated and processed by two more ConvLR blocks and a global average pooling layer. The final result is the three parameters $s_{v_{x}}$, $s_{v_{y}}$, and $s_{v_{pseudo}}$.

The MHA mechanism was initially proposed in the field of natural language processing (NLP) [13]. Later, Tsai et al. [14] explored leveraging MHA mechanism to reinforce a target modality with features from another data modality via learning the cross-modal attention. The following is the formulation of the attention output:

(28) $$\mathrm{Attention}\left(Q_{A},K_{B},V_{B}\right)=\mathrm{softmax}\left(\frac{Q_{A}K_{B}^{{}^{⊤}}}{\sqrt{d_{k}}}\right)V_{B}.$$

Following the definition of [14], $Q_{A}\in R^{d\times d_{A}}$ denotes the queries from the modality A, $K_{B}\in R^{d\times d_{B}}$ denotes the set of keys, and $V_{B}\in R^{d\times d_{B}}$ denotes the set of values from the modality B. Then, the information from modality B is passed to modality A by calculating the attention function in Equation (28). In this study, we leverage the MHA to model feature fusion via introducing one from another modality. As shown in Figure 2, taking acceleration data as an example, we reinforce its features via modeling the cross attention with angular velocity data by calculating attention output $\mathrm{Attention}(Q_{acc},K_{ang},V_{ang})$. A skip connection is also implemented, to sum up the output from the first 3×3 convolution layers and attention output.

### 4.2. Online Training Method

SLAM works well unless a sensor fails [26], which is a well-known problem statement. Therefore, our training system initially considers the SLAM outputs under ideal (reliable) conditions as ground truths to calculate the loss function between the output states of SLAM and EKF.

Furthermore, the iterative EKF estimation process in consecutive time steps is a kind of Markov decision process, which means that the current state is only related to the previous one, so our method focuses on optimizing each EKF estimation process. When the estimation performance of each EKF iteration is high, it will show high estimation accuracy in the entire iterative process. In order to evaluate the performance of each EKF estimation, the loss function is designed as follows:

(29) $$loss=\mathrm{MSE}\;(\;{\hat{x}}_{t+1|t+1}\;,\;x_{t+1}^{slam}),$$

where MSE is the mean squared error (MSE) function that expresses the bias of the estimated state by EKF compared to the state of SLAM at timestamp t+1. However, the performance of AGV localization depends on the two-dimensional (2D) position errors ($p_{x}$, $p_{y}$) and the heading angle (ψ) errors, so the loss function only needs to compute the mean squared error of $p_{x}$, $p_{y}$, and ψ calculated from the orientation state q. Thus, the loss function can be rewritten as

(30) $$loss=\mathrm{MSE}\;(\;{\left[\;\hat{p_{x}},\hat{p_{y}},\hat{\psi }\;\right]}_{t+1}^{T}\;,{\left[\;p_{x}^{slam},p_{y}^{slam},\psi^{slam}\;\right]}_{t+1}^{T}\;).$$

The initial state of each EKF iteration should be the same as the state of SLAM at the previous timestamp, so that the loss function can effectively express the error generated by each EKF iteration. Therefore, the input of trainable EKF that estimates the state at the next timestamp should be the state from SLAM at the current timestamp during training as follows:

(31) $${\hat{x}}_{t+1|t+1}=\mathrm{EKF}\left(x_{t}^{slam}\;,\;u_{t}\;,\;v_{t+1}^{cmd},\;P_{t}^{slam}\right),$$

which also considers the initial covariance $P_{t}$ of EKF as the current estimation covariance from SLAM.

**Figure 3:**
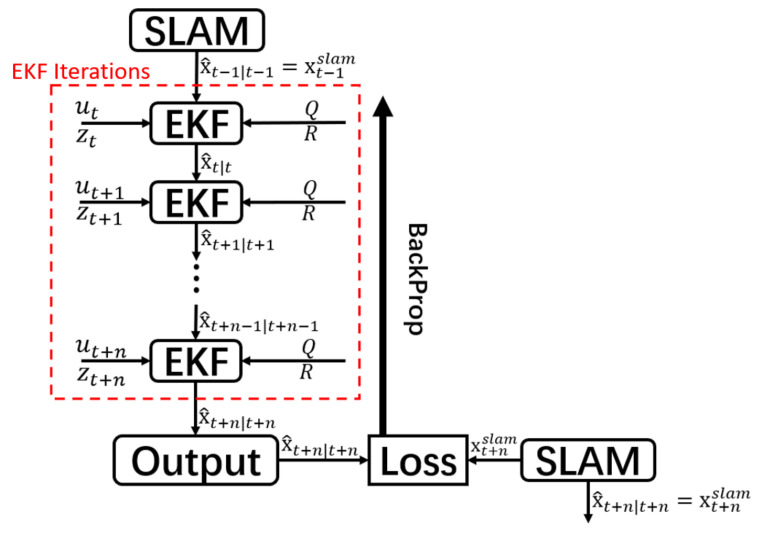
Structure and training flow of our network. The initial state of the EKF iterations is based on the previous SLAM state update at timestamp *t* − 1. *N* number of EKF iterations will be performed until the next update of the SLAM state at *t* + *n*. The loss is calculated between the SLAM state update and the final predicted output state of the *N* EKF updates. Then, backpropagation is performed on the basis of the calculated loss.

However, the frequency of SLAM is different from the frequency of IMU in real-time, so we cannot guarantee that the estimated state of each EKF iteration corresponds to the output state of each SLAM at the same time, which means that it is unable to regard the ground truth of EKF as SLAM at that time. Additionally, the frequency of IMU is usually higher than the frequency of SLAM, so to synchronize the output of EKF and SLAM, multiple EKF iterations can be trained by performing backpropagation [27] at once when the output state of SLAM is provided. The training structure is shown in Figure 3. The structure allows the system to optimize the covariance in real-time continuously. The training process begins with the MSE loss function (30), and then calculates the gradient of the loss function corresponding to each Q and R based on the derivative chain rule.

### 4.3. Implementation Details

This section introduces the settings and the implementation details of our algorithm. The whole self-adjusting method is implemented in Python with the PyTorch library [28] for training and inference, and the Robot Operating System (ROS) [29] was utilized for collecting sensor data.

The initial parameters of the EKF were set as follows prior to training. The initial system error covariance $P_{0}$ = $I_{10}$, which is the identity matrix 10×10. We set $\sigma_{a}$ = 0.01 m/s${}^{2}$ in (23), $\sigma_{\omega }$ = 0.01 rad/s${}^{2}$ in (24), $\sigma_{v}$ = 0.25 m/s in (25), and $\sigma_{v_{pseudo}}$ = 0.0225 m/s in (26). The adjustable parameters *s* are defined as $s_{a}$ = $s_{\omega }$ = $s_{v_{cmd}}$ = $s_{v_{pseudo}}$ = 0.01 in order to make initial covariance $\Sigma_{a}\approx \sigma_{a}^{2}$, $\Sigma_{\omega }\approx \sigma_{\omega }^{2}$, $\Sigma_{v_{cmd}}\approx \sigma_{v_{cmd}}^{2}$, and $\Sigma_{v_{pseudo}}\approx \sigma_{v_{pseudo}}^{2}$. We defined μ = 3 from (23) to (26), which allows for each covariance element to be $10^{3}$ times higher or smaller than its original values [12].

The Adam optimizer [30] was applied to update parameters with learning rate $10^{-3}$ and iterated once for each tandem EKF.

## 5. Experiments and Results

In this section, we present the robotic platform that was used for collecting data and testing a real-time SLAM failure scenario. Three evaluation metrics were used for evaluating the performance. The performance of our proposed localization architecture, the trainable quaternion-based EKF, was evaluated by comparing it with the EKF model without training. Additionally, various deep learning model architectures were compared to determine the most effective model.

### 5.1. Dataset

**Figure 4:**
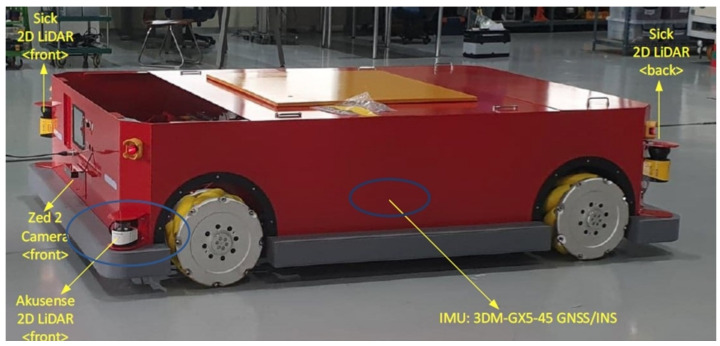
Robot setup used for data collection of the experiment.

The data collected to evaluate the proposed algorithm are based on the usage of a proprietary omniwheel robot platform of dimensions of 2.481×1.595 meters. The robotic system provides the current velocity of the robot, which is used as an input to the EK. The robot contains four 2D LiDARs (two in the front and two in the rear), two stereo cameras (one in the front and one in the rear), and an IMU. Figure 4 shows the robot setup.

Experiments were conducted across four different trajectories with total lengths of 66.46, 145.39, 103.42, and 78.62 meters, respectively. The trajectory path collected as a result of SLAM was used as the ground truth. For this work, the visual–LiDAR–inertial SLAM algorithm in [31] was used, as it provides a very accurate position and orientation. The dataset collected contained the SLAM output (ground truth position and orientation), the acceleration and angular velocity information, and IMU data for the EKF state estimation calculations.

Since the EKF state estimation was calculated at the origin of the IMU, all data points (SLAM and velocity) were transformed to the IMU’s origin. Furthermore, all data had to be transformed to the body frame coordinates of the omniwheel robot, where the x-axis runs along the length of the robot in the forward direction, the y-axis is 90 degrees anticlockwise, and the z-axis runs upward.

### 5.2. Experimental Setup

To evaluate the improvement of the proposed algorithm, the localization output of a traditional EKF was used as the experimental control data.

Our proposed system architecture was designed with the intention that the algorithm would be trained continuously while the robot is online; therefore, the data are collected in real time, and each data point is unique and independent. Taking this into account, we trained the algorithm on three sequence runs and performed the inference test on the fourth unseen sequence, with the data of each sequence only seen once during training and inference. This process was performed four times to run the inference test on all available sequences.

The EKF state estimation was then fused with the SLAM state during the inference mode while the system considered the SLAM positional data reliable, which is determined by the covariance values provided by the SLAM module. The time range when the SLAM positional data were unreliable was considered the SLAM failure period, at which point the SLAM state was no longer fused with the EKF estimation, and the output was purely relying on EKF.

To simulate a real-time SLAM failure scenario, 100 s time intervals within each individual trajectory were selected as the SLAM failure period. Both approaches, the traditional EKF and the proposed trainable EKF, had the same start and end times for the SLAM failure.

**Figure 5:**
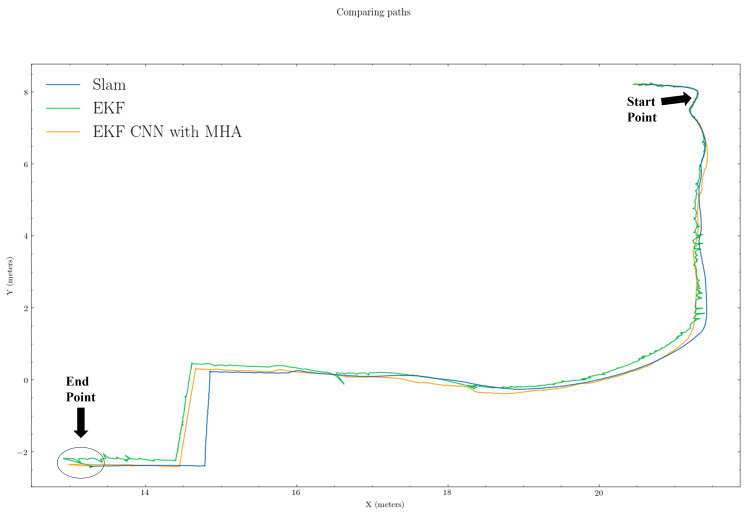
Estimated path comparison results for trajectory 1. Green, orange, and blue lines represent EKF, EKF+CNN with MHA, and ground truths, respectively.

**Figure 6:**
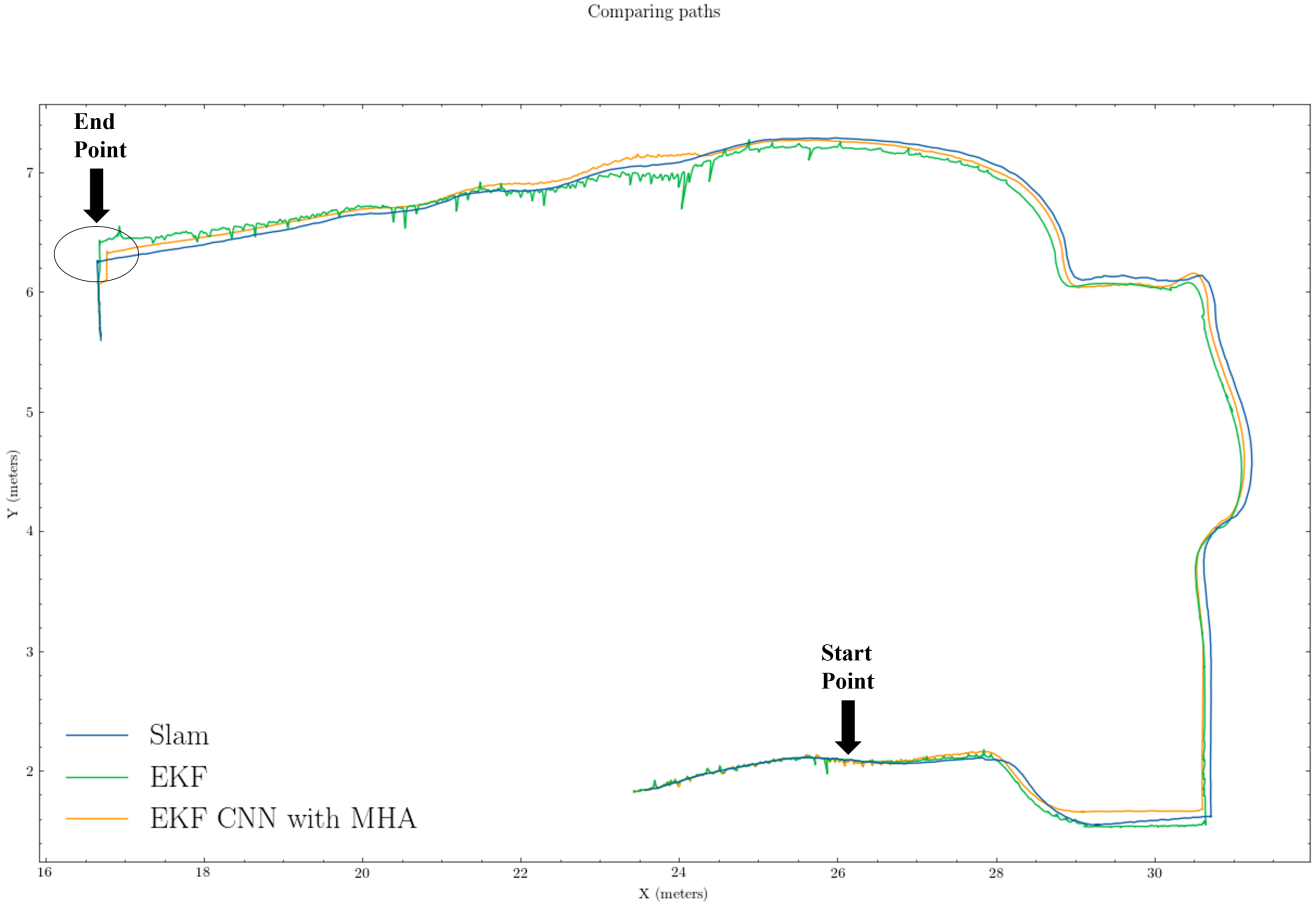
Estimated path comparison results for trajectory 4. Green, orange, and blue lines represent EKF, EKF+CNN with MHA, and ground truths, respectively.

In Figure 5 and Figure 6, the period from when SLAM failed (“Start Point”) to when SLAM recovered (“End Point”) were regarded as the SLAM failure period. The green and orange paths represent the estimated position from the untrained EKF and EKF CNN with MHA, respectively, while the blue path (ground truth) is from the SLAM outputs.

To evaluate the performance of our proposed algorithms, we considered the following three evaluation metrics for our experiments:

1. Position Error$\;(P_{e}):$
  The ratio of the position error to the total path length when SLAM fails.
  (32) $$P_{e}=\left(Last\;Position\;Error/Total\;Distance\right)*100$$
2. Rotation Error$\;(A_{e}):$
  Azimuth angle error relative to the total path length when SLAM fails.
  (33) $$A_{e}=Last\;Azimuth\;Angle\;Error/Total\;Distance$$
3. Average Mean Squared Error$\;(MSE_{avg})$:
  The overall average of the squares of the errors between ground truths (SLAM) and object to be assessed (estimated position and orientation) when SLAM fails.
  (34) $$MSE_{avg}=Mean\left(\;MSE\;\left(\;{\left[\;\hat{p_{x}},\hat{p_{y}},\hat{\psi }\;\right]}_{t+1}^{T}\;,{\left[\;p_{x}^{slam},p_{y}^{slam},\psi^{slam}\;\right]}_{t+1}^{T}\;\right)\;\right)$$

### 5.3. Result and Analysis

**Figure 7:**
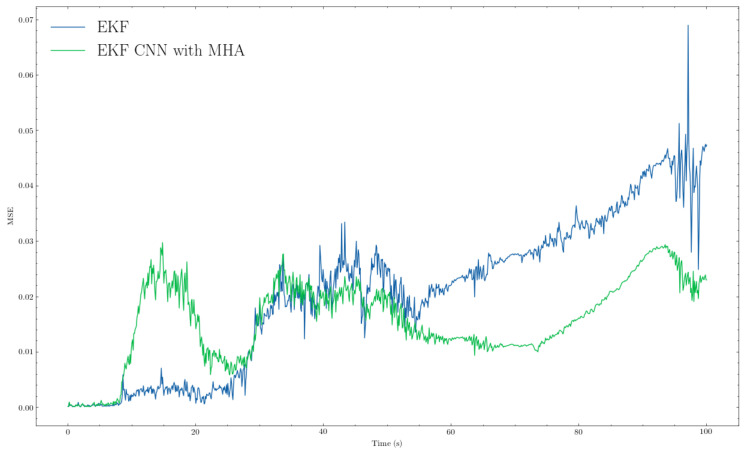
The Mean Squared Error (MSE) comparison between EKF and EKF+CNN with MHA for trajectory 1. Blue and green lines represent EKF and proposed method, respectively.

**Figure 8:**
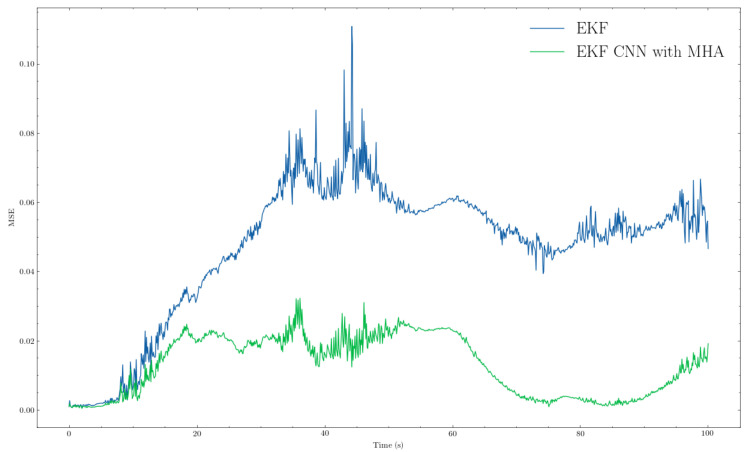
The Mean Squared Error (MSE) comparison between EKF and EKF+CNN with MHA for trajectory 3. Blue and green lines represent EKF and proposed method, respectively.

We present the comparison results of our proposed algorithm with those of the traditional method (i.e., EKF). From the trajectory comparisons in Figure 5 and Figure 6, we can see that the proposed EKF CNN with MHA (orange path) was more in line overall with less noise than the traditional EKF (green path). Furthermore, from Figure 7 and Figure 8, which provide the mean squared errors (MSE) during the failure period, we can see that the proposed method (green line) could achieve a lower MSE than the traditional EKF (blue line). From Figure 7 and Figure 8, the maximum MSE for the proposed method was 0.0297 and 0.0323, respectively, while the traditional EKF method reached 0.0690 and 0.1108 for the same trajectories.

Table 1 shows the full test results we collected and analyzed. As mentioned for each trajectory, an arbitrary interval of 100 s was chosen as the failure period, which could result in different failure lengths. Based on the provided results, it is clear that our proposed method overall outperformed the traditional EKF. In all cases, the position error $P_{e}$ and the rotation error $A_{e}$ were lower with the proposed method. In fact, the proposed method achieved 1.24765% for overall $P_{e}$ and 0.02785 deg/m for $A_{e}$. Similarly, the proposed method performed much better on the MSE metric.

Our trainable EKF CNN with MHA showed improved results over the traditional EKF estimation due to optimization of process and measurement errors through a combination of CNN inference, backpropagation, and gradient descent.

In Table 2, we also compared the performance of using different model architectures. For the implementation of EKF+CNN_1, we removed the MHA layer with skip connection and kept all the other components, and the EKF+CNN_2 was implemented by removing the last convolution layer of EKF+CNN_1. EKF+LSTM was also introduced to attempt to learn the temporal features from the data. As can be seen, eliminating the final convolution layer reduced Pe and Ae performance overall while somewhat lowering MSE, which EKF+LSTM also achieved. Nevertheless, the proposed architecture combining CNN and MHA showed considerable improvements in MSE and lowered $P_{e}$ a bit. The $A_{e}$ was still higher than that of EKF+CNN_1, but in the acceptable range.

### 5.4. Discussion

We demonstrated the advantages of our proposed trainable quaternion-based EKF with CNN and MHA based on the conducted experiments and analysis. Our proposed method could achieve 1.24765% in $P_{e}$, 0.02785 in $A_{e}$, and it significantly outperforms other model architectures in terms of MSE with 0.02713. The results also suggest that a trainable EKF, which can dynamically adjust process and measurement noise covariance matrices, can improve localization performance. Moreover, our proposed model architecture provides scope for fusing the inputs via reinforcing one modality by introducing features from another modality for the inputs of estimating covariance matrices’ parameters for EKF.

However, the online training cost of the proposed method can limit the overall performance. Furthermore, the time lag introduced by the online model training can be accumulated and cause deviations at the endpoints because of the limitations of the embedded computing system. Nonetheless, this issue can be solved by transmitting data to a more powerful server for online training. Furthermore, the future development of the AI-embedded platform will provide more power to achieve better online training performance. In conclusion, our results demonstrate that the deep learning model can be trained and provide predictions in an online training manner in a local integrated system.

## 6. Conclusions

In this paper, we designed a trainable and adjustable quaternion-based EKF algorithm with CNN and MHA for the sensor fusion of IMU-based localization and visual–LiDAR–inertial SLAM. Specifically, we developed an approach where the EKF-based localization system can provide a more accurate position estimation when SLAM failure occurs during a short time period. This was performed by tuning the process- and measurement-covariance matrices trained by CNN through backpropagation and further adjusting the velocity measurement covariances according to real-time IMU data through network inference. The approach leveraged the SLAM data as ground truths to compute the mean squared error of position and orientation estimated by the EKF while training.

For training and estimation, we designed a tandem EKF structure to adapt to the situation where real-time data from different sources were fused at different frequencies. Our proposed trainable EKF will be effective in dead-reckoning as a complementary process of SLAM when SLAM fails, which will enhance accuracy and stability in localization in complex and dynamic environments. Our future steps will focus on enhancing the structure of our proposed EKF CNN with MHA by performing multi-IMU fusion with multiple EKF modules, where each EKF leverages a unique IMU source.

## Figures and Tables

**Table 1:** Results for the four trajectory paths followed by evaluation metrics. Failure length: moving track length of robot during SLAM failure period; $P_{e}$: position error; $A_{e}$: rotation error; $MSE_{avg}$: average mean squared error.

Trajectory	Failure Length (m)	EKF			Proposed		
		$P_{e}$ (%)	$A_{e}$ (deg/m)	${\mathrm{MSE}}_{\mathrm{avg}}$	$P_{e}$ (%)	$A_{e}$ (deg/m)	${\mathrm{MSE}}_{\mathrm{avg}}$
1	18.19	1.64180	0.01320	0.02055	0.97901	0.00639	0.01580
2	23.92	3.56478	0.10087	0.11934	2.79777	0.04045	0.07454
3	22.94	1.00380	0.22513	0.04406	0.84346	0.01948	0.01294
4	24.81	0.38022	0.02335	0.00504	0.37035	0.04509	0.00523
Overall		1.64765	0.09064	0.04725	1.24765	0.02785	0.02713

**Table 2:** Results for the four trajectory paths followed by evaluation metrics. Failure length: moving track length of robot during SLAM failure period; $P_{e}$: position error; $A_{e}$: rotation error; $MSE_{o}$: overall mean squared error. CNN_1 is the proposed model architecture without the multi-head attention layer. CNN_2 is the same as CNN_1 but removes the last convolution layer. LSTM is implemented with a number of 2 layers and a hidden size of 256.

Model	$P_{e}$ (%)	$A_{e}$ (deg/m)	${\mathrm{MSE}}_{o}$
EKF	1.64765	0.09064	0.04725
EKF+CNN_1	1.25114	0.02111	0.04084
EKF+CNN_2	1.46609	0.03109	0.03696
EKF+LSTM	1.35288	0.03138	0.03573
Proposed	1.24765	0.02785	0.02713

## Data Availability

Not applicable.

## References

[1] Alatise M.B., Hancke G.P. (2020). A Review on Challenges of Autonomous Mobile Robot and Sensor Fusion Methods. IEEE Access.

[2] Ott F., Feigl T., Loffler C., Mutschler C. ViPR: Visual-Odometry-aided Pose Regression for 6DoF Camera Localization. Proceedings of the IEEE/CVF Conference on Computer Vision and Pattern Recognition Workshops.

[3] Zhang E., Masoud N. (2020). Increasing GPS Localization Accuracy With Reinforcement Learning. IEEE Trans. Intell. Transp. Syst..

[4] Lidow A., De Rooij M., Strydom J., Reusch D., Glaser J. (2019). GaN Transistors for Efficient Power Conversion.

[5] Zhao J. (2018). A Review of Wearable IMU (Inertial-Measurement-Unit)-based Pose Estimation and Drift Reduction Technologies. J. Phys. Conf. Ser..

[6] Thrun S., Burgard W., Fox D. (2005). Probability Robotics.

[7] Kalman R.E. (1960). A New Approach to Linear Filtering and Prediction Problems. J. Basic Eng..

[8] Malyavej V., Kumkeaw W., Aorpimai M. Indoor robot localization by RSSI/IMU sensor fusion. Proceedings of the 2013 10th International Conference on Electrical Engineering/Electronics, Computer, Telecommunications and Information Technology.

[9] Brossard M., Bonnabel S. Learning wheel odometry and IMU errors for localization. Proceedings of the 2019 International Conference on Robotics and Automation (ICRA).

[10] Yan Y., Zhang B., Zhou J., Zhang Y., Liu X. (2022). Real-Time Localization and Mapping Utilizing Multi-Sensor Fusion and Visual–IMU–Wheel Odometry for Agricultural Robots in Unstructured, Dynamic and GPS-Denied Greenhouse Environments. Agronomy.

[11] Jurado J., Kabban C.M.S., Raquet J. (2019). A regression-based methodology to improve estimation of inertial sensor errors using Allan variance data. Navig. J. Inst. Navig..

[12] Brossard M., Barrau A., Bonnabel S. (2020). AI-IMU dead-reckoning. IEEE Trans. Intell. Veh..

[13] Vaswani A., Shazeer N., Parmar N., Uszkoreit J., Jones L., Gomez A.N., Kaiser Ł., Polosukhin I. (2017). Attention is all you need. Adv. Neural Inf. Process. Syst..

[14] Tsai Y.H.H., Bai S., Liang P.P., Kolter J.Z., Morency L.P., Salakhutdinov R. Multimodal transformer for unaligned multimodal language sequences. Proceedings of the 57th Annual Meeting of the Association for Computational Linguistics.

[15] Akhlaghi S., Zhou N., Huang Z. Adaptive adjustment of noise covariance in Kalman filter for dynamic state estimation. Proceedings of the 2017 IEEE Power & Energy Society General Meeting.

[16] Hu G., Gao B., Zhong Y., Gu C. (2020). Unscented kalman filter with process noise covariance estimation for vehicular ins/gps integration system. Inf. Fusion.

[17] Hornik K., Stinchcombe M., White H. (1989). Multilayer feedforward networks are universal approximators. Neural Netw..

[18] Haarnoja T., Ajay A., Levine S., Abbeel P. (2016). Backprop kf: Learning discriminative deterministic state estimators. Adv. Neural Inf. Process. Syst..

[19] Song F., Li Y., Cheng W., Dong L., Li M., Li J. (2022). An Improved Kalman Filter Based on Long Short-Memory Recurrent Neural Network for Nonlinear Radar Target Tracking. Wirel. Commun. Mob. Comput..

[20] Gao X., Luo H., Ning B., Zhao F., Bao L., Gong Y., Xiao Y., Jiang J. (2020). RL-AKF: An adaptive kalman filter navigation algorithm based on reinforcement learning for ground vehicles. Remote Sens..

[21] Wu F., Luo H., Jia H., Zhao F., Xiao Y., Gao X. (2020). Predicting the noise covariance with a multitask learning model for Kalman filter-based GNSS/INS integrated navigation. IEEE Trans. Instrum. Meas..

[22] Feng K., Li J., Zhang X., Shen C., Bi Y., Zheng T., Liu J. (2017). A new quaternion-based Kalman filter for real-time attitude estimation using the two-step geometrically-intuitive correction algorithm. Sensors.

[23] Kok M., Hol J.D., Schön T.B. (2017). Using inertial sensors for position and orientation estimation. arXiv.

[24] Mochnac J., Marchevsky S., Kocan P. Bayesian filtering techniques: Kalman and extended Kalman filter basics. Proceedings of the 2009 19th International Conference Radioelektronika.

[25] NGOC T.T., KHENCHAF A., COMBLET F. Evaluating Process and Measurement Noise in Extended Kalman Filter for GNSS Position Accuracy. Proceedings of the 2019 13th European Conference on Antennas and Propagation (EuCAP).

[26] Khairuddin A.R., Talib M.S., Haron H. Review on simultaneous localization and mapping (SLAM). Proceedings of the 2015 IEEE International Conference on Control System, Computing and Engineering (ICCSCE).

[27] LeCun Y., Touresky D., Hinton G., Sejnowski T. A theoretical framework for back-propagation. Proceedings of the 1988 Connectionist Models Summer School.

[28] Paszke A., Gross S., Massa F., Lerer A., Bradbury J., Chanan G., Killeen T., Lin Z., Gimelshein N., Antiga L. (2019). Pytorch: An imperative style, high-performance deep learning library. Adv. Neural Inf. Process. Syst..

[29] Stanford Artificial Intelligence Laboratory Robotic Operating System. https://www.ros.org.

[30] Kingma D.P., Ba J. (2014). Adam: A method for stochastic optimization. arXiv.

[31] Nam D.V. (2022). Robust Multi-Sensor Fusion-based SLAM using State Estimation by Learning Observation Model. Ph.D. Thesis.

